# Digital tracking of girls exposed to community led alternative rites of passage to prevent female genital mutilation/cutting, and child, early and forced marriages in Kenya: a longitudinal study

**DOI:** 10.3389/frph.2025.1445504

**Published:** 2025-05-20

**Authors:** David Kawai, Bernard Mbogo, Yvonne Opanga, Samuel Muhula, Tammary C. Esho, Hilke Conradi, Viola J. Rutto, Denge Lugayo, Dennis J. Matanda

**Affiliations:** ^1^ Amref Health Africa, Nairobi, Kenya; ^2^ Amref International University, Nairobi, Kenya; ^3^ Amref Health Africa, Leiden, Netherlands; ^4^ Population Council, Nairobi, Kenya

**Keywords:** alternative rite of passage, female genital mutilation/cutting, child marriage, Kajiado county, Kenya

## Abstract

**Introduction:**

Female genital mutilation/cutting (FGM/C) and child marriage (CEFM) are harmful practices that are a human rights violation. For decades, many interventions have been implemented to end these practices. One such intervention is the Alternative Rite of Passage (ARP), which allows girls to go through a meaningful rite of passage without the cut. The ARPs have come under scrutiny due to a lack of data to show how effective ARPs have been. This study aimed to establish the effect of the Community-Led Alternative Rite of Passage (CL-ARP) model on incidences of FGM/C, CEFM and keeping girls and young women in school.

**Methods:**

The study adopted a longitudinal design where girls and young women were enrolled into the CL-ARP programme and later followed up for over three years to assess the effectiveness of the CL-ARP model in preventing incidences of FGM/C, CEFM and keeping girls in school. A total of 2,647 girls aged 10–23 years who resided in Kajiado County were recruited and followed up post-exposure to CL-ARP. Data analysis involved conducting descriptive and logistic regression analyses.

**Results:**

The CL-ARP programme kept 98% of girls free of FGM/C, 99% free of CEFM and 98% kept in school. 41 cases of FGM/C, 12 cases of CEFM and 48 cases of school dropouts were reported three years post-exposure. Girls who underwent FGM/C had been kept free of FGM/C for an average of 39.5 months, those who experienced CEFM had been kept free of CEFM for an average of 40.2 months, and those who dropped out of school had been kept in school for an average of 38.5 months. Girls and young women who experienced instances of threats/violence were more likely to experience FGM/C, CEFM and drop out of school than those who had not.

**Conclusions:**

The CL-ARP programme was successful in keeping the majority of girls and young women free of FGM/C and CEFM, and retained in school post-enrollment. Reported cases of FGM/C, CEFM and school dropouts underline the importance of considering other contextual factors such as gender-based violence that may continue to put girls and young women at risk despite embracing CL-ARP.

## Introduction

Female Genital Mutilation/Cutting (FGM/C) is defined by the World Health Organization (WHO) as the partial or complete removal of the female external genitalia or any other injury to the female genital organs for non-medical reasons (1). Across the globe, over 230 million girls and women have been subjected to FGM/C and more than 70 million girls aged 0-14 years are estimated to be at risk of FGM/C annually (2, 3). Regional statistics for Africa show that FGM/C is practised in 28 countries with geographical spread in the East, North, West, Central, and the Horn of Africa. It is estimated that across these 28 countries, more than 144 million women and girls aged ten years and above have undergone the practice (3).

At a national level, Kenya's most recent prevalence of FGM/C among girls and women of reproductive age 15–49 years was 15% in 2022 (4). Nationally representative data collected by the Kenya Demographic and Health Survey (KDHS) for over two decades indicates that the national prevalence of FGM/C has been decreasing. From the KDHS wave of 1998 where FGM/C prevalence was 38%, the prevalence dropped to 32% in 2003, 27% in 2009, 21% in 2014% and 15% in 2022 (4–8). While the national estimates paint a success story in the reduction of FGM/C in Kenya, there exist significant within-country variations, with prevalence ranging from less than 1% in counties in the Western region to a high of over 83% in counties in the North-Eastern region (4). Among the Maasai ethnic group who are the focus of this study and predominantly live in Kajiado and Narok counties in the Rift-Valley region, the FGM/C prevalence is estimated to be 53% (4).

In Kenya, the practice of FGM/C is performed mostly on girls between the ages of 12 and 18 years though studies have shown that the age at cutting has been decreasing with girls cut secretly at younger ages between 7 and 12 years (9–11). In the majority of communities that practice FGM/C, the practice has over the years been culturally associated with readiness for marriage. Even though the minimum legal age for marriage in Kenya is 18 years, and the Children's Act of 2001 outlaws child, early or forced marriage, many marriages are not officially registered or are performed under customary or Islamic law, which has no age restriction. In this context, FGM/C could be a precursor to other harmful practices including child, early and forced marriage, and dropping out of school (12, 13).

Among the key drivers of FGM/C are the sociocultural factors reinforced by community pressure to conform and the threat of stigma (14). Even though there are specific factors that motivate individual communities in Kenya to continue practicing FGM/C, there are several unifying rationales and beliefs. For example, among the Maasai, Samburu and Abagusii ethnic groups, the practice is sanctioned on young girls as a rite of passage signifying the transition from “childhood” to “womanhood” (15). FGM/C in these communities is often conducted as part of an initiation into womanhood or a maturity ritual. Specifically, among the Maa community that brings together the Maasai and the Samburu, this rite of passage or initiation into womanhood by undergoing FGM/C is a key lifetime ceremony for young girls. The practice has future ramifications including acceptance by community members and peers, and marriage prospects with those who have undergone the practice likely to fetch a higher bride price in form of cows to the bride's family (16).

Many interventions have been rolled out in various contexts including intervention in public health, social and gender norms change, community education, legal and policy frameworks among others (17). One such intervention has been the alternative rite of passage (ARP) which refers to an alternative to FGM/C that retains cultural rituals and ceremonies in the transition of girls to womanhood that has been implemented in communities which have practiced FGM/C as a rite of passage into adulthood. Since 2009, Amref Health Africa has been implementing the community-led alternative rite of passage (CL-ARP) as an intervention aimed at ending harmful traditional practices including FGM/C and child, early and forced marriages (CEFM). Even though there has been some evidence suggesting success of CL-ARP intervention activities (18–20), there is still a dearth of knowledge regarding the effectiveness of the approach in protecting young women and girls from harmful traditional practices including FGM/C and CEFM (21). In one of the studies conducted to evaluate the impact of CL-ARP, findings showed that CL-ARP was associated with an increase in the years of schooling for girls by 2.5 years, and declines in FGM/C (by 24.2%), health costs (USD 600,000 per year), child marriage (by 4.9%) and teenage pregnancies (by 6.3%) (22). A key limitation of this study is that the data used was derived from a national survey (KDHS) which is a secondary dataset not specifically collected to evaluate the effectiveness of the ARP model. Significantly, there were other confounding factors including implementation of other FGM/C interventions by other partners and therefore not possible to directly link the observed changes to the ARP model alone. Understanding the effectiveness of the CL-ARP is critical given the popularity of this approach and the interest among programme implementers and policymakers on what works to end FGM/C. The fact that FGM/C is significantly influenced by social norms and the CL-ARP is an intervention expected to facilitate change in such norms leading to change in behaviour, understanding the effectiveness of the CL-ARP therefore adds value to the theoretical discourse of FGM/C as a practice informed by the social norm theory.

Given the noted limitations of studies that have attempted to evaluate the effectiveness of the ARP model, this study aimed to ascertain the effectiveness of the CL-ARP model with data collected from a selected cohort of girls. The study followed up girls who had gone through the CL-ARP process—details can be found in the published study protocol (23). Over the years, follow up of girls who have graduated through the CL-ARP process has not been consistent especially after the graduation ceremony. This challenge was further compounded by the emergence of the COVID-19 pandemic where community meetings became impossible and physical tracking of the girls was negatively affected (24). These challenges called for an innovative way to follow up the girls in such crisis situations. Consequently, Amref Health Africa deployed a digital tracking tool to monitor girls who have gone through CL-ARP as a means to end FGM/C, CEFM and keep them in school until they attain the age of 24 years. The main objective of the study was to determine the effect of CL-ARP on FGM/C, CEFM and school attendance using a digital tracking mechanism.

## Materials and methods

### Study design

The study adopted a longitudinal design to assess the effectiveness of CL-ARP model in preventing FGM/C, CEFM and keeping girls in school. Girls aged 10–18 years were enrolled into the CL-ARP programme and after being exposed to the CL-ARP interventions, they were followed for over three years to assess whether the CL-ARP model was successful in preventing incidences of FGM/C and CEFM and keeping girls in school.

### Study setting

This study was implemented in Kajiado County in Kenya where Amref Health Africa has been conducting CL-ARPs. Kajiado County is predominantly inhabited by the Maasai ethnic group whose main economic activities include tourism and agriculture, especially livestock rearing. The Maasai community has traditionally practiced FGM/C—terming it as a rite of passage that symbolizes a girl's transition from childhood to womanhood (25). This transition from childhood to womanhood is sometimes associated with CEFM and girls dropping out of school. The significance of the practice is to enable the young girl gain respect in the community, be educated about her role in society and prepared for her next phase in life including preparation for marriage (26). Once in a year, especially during school holidays, FGM/C takes place usually between the ages of 12–14 years (27). The most common type of FGM/C among the Maasai community is Type II (excision) (28). Data from the 2022 Kenya demographic and health survey indicates the FGM/C prevalence among the Maasai to be at 53% having declined from 78% in 2014 (4).

### Study participants

The study targeted girls and young women eligible for training and graduating from the CL-ARP programme in Kajiado county. Specifically, the inclusion criteria at enrollment included girls aged between 10 and 18 years and were willing to participate in the CL-ARP programme activities, girls who had not undergone FGM/C, were not pregnant, not married at the time of recruitment, and were in school at the point of enrollment. Young women who did not consent to be part of the study, minors who did not assent to be part of the study, and minors whose parents did not consent to be part of the study were excluded from the study.

### CL-ARP intervention activities

The CL-ARP model is a community-led cultural alternative to FGM/C that retains cultural rituals and ceremonies in the transition to womanhood, whilst replacing the harmful “cut” by sexual and reproductive health rights education and the promotion of girls' education. This process works through structured community entry and intergenerational community dialogues with various community members including men and boys who are actively engaged in addressing norms, attitudes and behaviors underlying FGM/C and other forms of sexual violence. They also address harmful traditional practices including child and early marriage. After this initial engagement with the whole community and buy in to the alternative process, the real initiation process with the girls begins. This follows the tripartite sequence of separation, transition and assumption. First, the girls undergo a separation process whereby they are taken into seclusion, a safe space within the community, where they will reside during this whole process. The transition process involves training of girls on a variety of topics including sexual and reproductive health, the dangers of FGM/C, early marriage, teenage pregnancy, life skills and gender issues, self-esteem, life skills and children's rights. It culminates in a graduation which takes the original traditional format that involves the girls, parents, women leaders, elders and other community leaders. The CL-ARP period consists of 8–48 months and only when a community is ready does the graduation ceremony takes place. The assumption involves the new initiates taking up new roles as envisaged in the traditional rites of passage. After the public ceremony, the girls are registered to a digital platform and tracked. The digital tracking involves collection of longitudinal data from the girls including their FGM/C, teenage pregnancy and child marriage statuses. Data is also collected on socio-economic, educational and health outcome indicators of the girls.

### Data collection

Data collection was conducted using a mobile app developed by Amref, Taroworks and Salesforce. Girls enrolled into the CL-ARP programme were followed up by trained community health volunteers and teachers who administered questionnaires about their wellbeing, safety, and if they faced any threats of FGM/C, CEFM, teenage pregnancies, dropping out of school or any other forms of abuse or violation of their rights.

The app has a case management module where FGM/C, teenage pregnancies, CEFM and school dropout cases are reported. The reported cases are immediately shared with the established local government/community structures for action. Girls are visited every 6 months and asked questions about their wellbeing. The check-ins also follow up on girls' various socio-economic aspects of their lives including career progression. Through these check-ins and monitoring of the girls, it was possible to generate data on whether the girls stayed protected from FGM/C and CEFM, retained in school, and document any other risks they faced including instances of threats and violence. The identity of girls participating in the study was protected through anonymization and girls guaranteed utmost confidentiality of the data collected. A check-in list with detailed questions that were used to generate data can be availed upon request.

### Data analysis

Study data was electronically transmitted to servers domiciled at Amref headquarters in Nairobi. The transmitted data was first downloaded in an Excel format then exported to a statistical software (STATA) for analysis. Key variables that were tracked included: (i) Incidence of FGM/C—number of new cases of FGM/C within the cohort; (ii) Incidence of teenage pregnancies—number of new cases of teenage pregnancies within the cohort; (iii) Incidence of CEFM—number of new cases of early child marriages within the cohort; (iv) Educational attainment—education level of girls and young women within the cohort and cases of school dropouts, (v) experiences of violence—instances/threats including physical abuse, expulsion from home and ridicule; and (vi) State of mind—how girls and young women generally perceived their mental wellbeing.

Analysis involved conducting descriptive, bivariate and multivariate analyses using STATA. Descriptive statistics were generated to show background characteristics of respondents and group proportions across key variables. At bivariate level, the analysis included assessing associations between dependent variables (FGM/C, CEFM and school attendance) and an independent variable (Model I). At multivariate level, variables at bivariate level were subjected to regression models (Model II).

## Results

### Background characteristics of respondents

Table 1 summarizes the background characteristics of girls and young women who participated in the study. It is important to note that while girls were enrolled at the ages of 10–18 years, some were aged over 18 years at the time of the interviews (post-exposure to CL-ARP). Majority of study participants were of ages 15–17 years (47%) followed by those aged 10–14 years (33%) while fewer were aged between 18 and 23 years (20%). Half of the study participants were in lower secondary education level while a third were in upper primary (32%). Most of the girls and young women who participated in this study had career dreams and aspirations (84%).

**Table 1:** Background characteristics.

Characteristics	Percent (%)	*N*
Age		
10–14 years	33.1	746
15–17 years	46.6	1,052
18–23 years	20.3	458
Education level		
Lower primary (Grades 1, 2 & 3)	4.4	114
Upper primary (Grades 4, 5 & 6)	32.3	838
Lower secondary (Grades 7, 8 & 9)	49.8	1,293
Senior school (Grades 10, 11 & 12)	12.0	311
Tertiary (TVET, college & university)	1.6	42
Career dreams and aspirations		
Without dreams and aspirations	16.4	434
With dreams and aspirations	83.6	2,213
Total	**100**.**0**	**2,647**

Bold values indicates number of girls and young women who participated in the study.

### Incidences of FGM/C, CEFM and school dropout

To assess cases of FGM/C, CEFM and school dropout, girls and young women who had gone through the CL-ARP programme were asked by community health volunteers during the 6-months check-ins whether they had experienced FGM/C, CEFM or were out of school. Table 2 shows that over 98% of girls who were enrolled into the CL-ARP programme did not undergo FGM/C, CEFM or drop out of school after more than three years post-exposure. However, 15 cases of FGM/C, 12 cases of CEFM and 48 cases of girls dropping out of school were reported. For girls who either underwent FGM/C, experienced CEFM or dropped out of school, the CL-ARP programme managed to keep them free of FGM/C for an average of 39.5 months, free of CEFM for an average of 40.2 months, and kept them in school for an average 38.5 months.

**Table 2:** Incidences of FGM/C, CEFM, and current school attendance of girls and young women.

Characteristics	Percent (%)	*N*	Months saved from FGM/C, CEFM or kept in school
FGM/C incidence			
Yes	0.6	15	Mean (39.5), SD (18.66)
No	99.4	2,563	N/A
CEFM incidence			
Yes	0.5	12	Mean (40.2), SD (3.56)
No	99.5	2,634	N/A
Current school attendance			
Not attending school	1.9	48	Mean (38.5), SD (16.69)
Attending school	98.1	2,415	N/A

SD, standard deviation.

### Threats and violence against girls and young women and their state of mind

One of the objectives of the CL-ARP programme was to document instances of threats and violence against girls and women in the intervention sites and to link such cases to relevant authorities for support. During the check-ins, girls and young women enrolled into the CL-ARP programme were asked whether they felt safe in their community, whether they had experienced instances of threats or violence, whether they had been ridiculed, forced to run away from home, and their general state of mind. Findings in Table 3 show that majority of girls and young women (over 90%) in the programme did not experience instances of threats, violence or forced to run away from home, and felt that their mental wellbeing was good. Nonetheless, 97 girls and young women felt unsafe living in their community, 82 experienced instances/threats of FGM/C, 8 experienced instances/threats of physical abuse, 7 experienced instances/threats of expulsion from home, 12 experienced instances/threats of forced marriage, 23 were ridiculed, and 11 experienced instances where they were forced to run away from home, and 274 of girls and young women felt that their state of mind was bad.

**Table 3:** Experiences of threats, violence and state of mind of girls and young women.

Characteristics	Percent (%)	*N*
Feels safe		
No	3.7	97
Yes	96.3	2,549
Instances/threats of FGM/C		
Yes	3.1	82
No	96.9	2,564
Instances/threats of physical abuse		
Yes	0.3	8
No	99.7	2,638
Instances/threats of expulsion from home		
Yes	0.3	7
No	99.7	2,639
Instances/threats of forced marriage		
Yes	0.5	12
No	99.5	2,634
Instances of ridicule		
Yes	0.9	23
No	99.1	2,623
Instances of running away from home		
Yes	0.4	11
No	99.6	2,635
State of mind		
Bad	10.4	274
Good	89.6	2,372

### Associations between experiences of threats/violence and FGM/C

Table 4 shows findings from bivariate (Model I) and multivariate (Model II) logistic regression analysis, assessing the association between girls' and young women's experiences of threats/violence and FGM/C. In model I, girls and young women who experienced instances/threats of physical abuse had higher odds of experiencing FGM/C (COR = 27.33; *p* < 0.003), as well as those who experienced instances/threats of expulsion from home (COR = 41.04; *p* < 0.001), those who experienced instances/threats of forced marriage (COR = 32.82; *p* < 0.002), those who were ridiculed (COR = 9.60; *p* < 0.034) and those who experienced instances of running away from home (COR = 20.48; *p* < 0.006) compared to their counterparts who did not experience instances of threats/violence. Girls and young women who had career dreams and aspirations were less likely to experience FGM/C (COR = 0.14; *p* < 0.001) as compared to those who did not have career dreams and aspirations.

**Table 4:** Crude and adjusted odds ratios from bivariate and multivariate logistic regression models showing factors associated with FGM/C.

Characteristics	Model I			Model II		
	COR	95% CI	*P*-value	AOR	95% CI	*P*-value
Education level						
Primary®	1.00			1.00		
Lower secondary	3.06	(0.66, 14.21)	0.153	2.97	(0.61, 14.47)	0.179
Senior school and above	3.68	(0.61, 22.12)	0.155	3.59	(0.58, 22.23)	0.169
Dreams and aspirations						
Without dreams and aspiration ®	1.00			1.00		
With dreams and aspirations	0.14	(0.05, 0.40)	0.000	0.11	(0.03, 0.33)	0.000
Instances/threats of FGM/C						
No®	1.00			1.00		
Yes	2.66	(0.34, 20.70)	0.349	0.16	(0.01, 27.15)	0.480
Instances/threats of physical abuse						
No®	1.00			1.00		
Yes	27.33	(3.07, 243.28)	0.003	57.22	(0.31, 10,511.29)	0.128
Instances/threats of expulsion from home						
No®	1.00			1.00		
Yes	41.04	(4.29, 392.54)	0.001	8.42	(0.01, 35,156.10)	0.616
Instances/threats of forced marriage						
No®	1.00			1.00		
Yes	32.82	(3.58, 300.69)	0.002	10.76	(0.01, 27,090.62)	0.552
Instances of ridicule						
No®	1.00			1.00		
Yes	9.60	(1.19, 77.53)	0.034	1.15	(0.01, 289.80)	0.960
Instances of running away from home						
No®	1.00			1.00		
Yes	20.48	(2.39, 175.68)	0.006	1.33	(0.01, 282.36)	0.916
State of mind						
Bad®	1.00			1.00		
Good	1.39	(0.18, 10.65)	0.754	1.81	(0.21, 15.81)	0.593

COR, crude odds ratio; AOR, adjusted odds ratio; ®, reference category; CI, confidence intervals.

In the multivariate analysis (Model II), association between girls' and young women's experiences of threats/violence and FGM/C became less significant. However, having career dreams and aspirations remained a significant factor associated with experiencing FGM/C (AOR = 0.11; *p* < 0.001).

### Associations between experiences of threats/violence and CEFM

Findings from bivariate and multivariate logistic regression analyses examining the association between girls' and young women's experiences of threats/violence and CEFM are shown in Table 5. In model I, girls and young women who experienced instances/threats of FGM/C had higher odds of experiencing CEFM (COR = 75.58; *p* < 0.001) as well as those who experienced instances/threats of physical abuse (COR = 73.50; *p* < 0.001), those who experienced instances/threats of expulsion from home (COR = 368.00; *p* < 0.001), those who were ridiculed (COR = 25.81; *p* < 0.004) and those who were forced to run away from home (COR = 55.07; *p* < 0.001) compared to their counterparts who did not experience instances of threats/violence.

**Table 5:** Crude and adjusted odds ratios from bivariate and multivariate logistic regression models showing factors associated with CEFM.

Characteristics	Model I			Model II		
	COR	95% CI	*P*-value	AOR	95% CI	*P*-value
Feels safe						
No®	1.00			1.00		
Yes	0.15	(0.02, 1.29)	0.084	0.90	(0.04, 20.34)	0.950
Instances/threats of FGM/C						
No®	1.00			1.00		
Yes	75.58	(13.57, 421.08)	0.000	40.87	(4.83, 345.77)	0.001
Instances/threats of physical abuse						
No®	1.00			1.00		
Yes	73.50	(7.43, 727.02)	0.000	4.61	(0.19, 113.99)	0.351
Instances/threats of expulsion from home						
No®	1.00			1.00		
Yes	368.00	(47.83, 2,831.34)	0.000	201.83	(4.08, 9,992.01)	0.008
Instances of ridicule						
No®	1.00			1.00		
Yes	25.81	(2.86, 232.82)	0.004	0.08	(0.01, 111.49)	0.495
Instances of running away from home						
No®	1.00			1.00		
Yes	55.07	(5.77, 525.93)	0.000	0.63	(0.01, 723.54)	0.899
State of mind						
Good®	1.00			1.00		
Bad	0.20	(0.04, 1.12)	0.068	0.78	(0.07, 9.28)	0.842

COR, crude odds ratio; AOR, adjusted odds ratio; ®, reference category; CI, confidence intervals.

In the multivariate analysis (Model II), only girls' and young women's experiences of instances/threats of FGM/C and expulsion from home remained significantly associated with CEFM.

### Associations between experiences of threats/violence and school attendance

 Table 6 presents bivariate and multivariate logistic regression analyses examining the association between girls' and young women's experiences of threats/violence and their school attendance. Bivariate analyses (Model I) showed that girls and young women who experienced instances/threats of FGM/C were less likely to be out of school (COR = 0.12; *p* < 0.001) as well as those who experienced instances/threats of expulsion from home (COR = 0.05; *p* < 0.001), and those who experienced instances/threats of forced marriage (COR = 0.03; *p* < 0.001) compared to their counterparts who did not experience instances of threats/violence. Additionally, girls and young women in the age category 15–23 years had lower odds of dropping out of school (COR = 0.21; *p* < 0.004) as compared to the younger ones aged 9–14 years. Girls and young women who felt unsafe living in their community (COR = 18.97; *p* < 0.001) and those who felt that their state of mind was bad (COR = 6.15; *p* < 0.001) had higher odds of dropping out of school compared to those who felt safe and were of the view that their state of mind was good.

**Table 6:** Crude and adjusted odds ratios from bivariate and multivariate logistic regression models showing factors associated with current school attendance.

Characteristics	Model I			Model II		
	COR	95% CI	*P*-value	AOR	95% CI	*P*-value
Age						
9–14 years®	1.00			1.00		
15–23 years	0.21	(0.08, 0.61)	0.004	0.25	(0.09, 0.73)	0.011
Dreams and aspirations						
Without aspirations and dreams®	1.00			1.00		
With aspirations and dreams	1.77	(0.77, 4.06)	0.177	1.05	(0.40, 2.72)	0.927
Feels safe						
No®	1.00			1.00		
Yes	18.97	(9.51, 37.85)	0.000	12.91	(4.24, 39.30)	0.000
Instances/threats of FGM/C						
No®	1.00			1.00		
Yes	0.12	(0.05, 0.27)	0.000	0.31	(0.11, 0.85)	0.022
Instances/threats of expulsion from home						
No®	1.00			1.00		
Yes	0.05	(0.01, 0.24)	0.000	0.41	(0.04, 4.89)	0.484
Instances/threats of forced marriage						
No®	1.00			1.00		
Yes	0.03	(0.01, 0.10)	0.000	0.15	(0.03, 0.82)	0.029
Instances of ridicule						
No®	1.00			1.00		
Yes	0.34	(0.04, 2.60)	0.298	3.56	(0.30, 42.26)	0.314
State of mind						
Good®	1.00			1.00		
Bad	6.15	(3.20, 11.84)	0.000	1.09	(0.35, 3.37)	0.879

COR, crude odds ratio; AOR, adjusted odds ratio; ®, reference category; CI, confidence intervals.

In the multivariate analysis (Model II), girls' and young women's experiences of instances/threats of FGM/C (AOR = 0.31; *p* < 0.022) and forced marriage (AOR = 0.15; *p* < 0.029) remained significantly associated with dropping out of school. Other variables that remained significantly associated with school attendance included age and safety whereby girls and young women aged 15–23 years had lower odds of dropping out of school (AOR = 0.25; *p* < 0.011) compared to those aged 9–14 years. Girls and young women who felt unsafe living in their community (AOR = 12.91; *p* < 0.001) had higher odds of dropping out of school compared to those who felt safe.

## Discussion

This study sought to determine the effect of CL-ARP on FGM/C, CEFM and school attendance among girls and young women who had graduated from the CL-ARP programme. Study findings showed that over 98% of girls who were enrolled into the CL-ARP programme managed to stay free of FGM/C, CEFM and stayed in school after more than three years of exposure. For girls who either underwent FGM/C, experienced CEFM or dropped out of school, the CL-ARP programme managed to keep them free of FGM/C, CEFM and in school for a substantial period of time before they eventually experienced these negative life events. These findings point to the importance of community-based programmes such as CL-ARP and their potential in addressing harmful traditional practices such as FGM/C and CEFM. In the literature, a good example of a similar programme that embraced the community-led approach is the Tostan programme (29). The programme uses community-based education, community dialogue and debate, and public declaration to facilitate behaviour change including FGM/C abandonment. An evaluation of the effectiveness of the programme showed greater success with improvements in knowledge and attitudes toward FGM/C among women and men (29). Findings from the current study highlight the fact that an ARP intervention using a community-led model has a greater impact in protecting girls and young women from harmful traditional practices such FGM/C and CEFM while keeping them in school. While the CL-ARP model showed impressive results in protecting girls and young women from FGM/C and CEFM and keeping them in school, cases of FGM/C, CEFM and school dropouts were reported. This underlines the importance of considering other contextual factors including lack of money for school fees that may continue to put girls and young women at risk despite embracing CL-ARP (14, 30, 31).

An important objective of the CL-ARP programme was to document instances of threats and violence against girls and women and to link such cases to relevant authorities for support. While majority of girls and young women in the programme did not experience instances of threats, violence or forced to run away from home, and felt that their general mental wellbeing was good, there were reported cases of girls and young women who experienced instances of threats/violence and felt that their state of mind was bad. This study examined whether there was an association between girls' and young women's experiences of threats/violence and FGM/C, CEFM and school dropout. For the case of FGM/C, while girls and young women who experienced instances/threats of physical abuse, expulsion from home, forced marriage, were ridiculed and forced to run away from home had higher odds of experiencing FGM/C, this association became less significant when controlled against other factors. Significantly, having career dreams and aspirations remained a significant factor in protecting girls and young women from undergoing FGM/C. Regarding the association between girls' and young women's experiences of threats/violence and CEFM, the key finding was that girls' and young women's experiences of instances/threats of FGM/C and expulsion from home was a consistent significant risk factor for CEFM. With regards to the association between girls' and young women's experiences of threats/violence and their school attendance, a key finding was that girls and young women who felt unsafe living in their community had higher odds of dropping out of school compared to those who felt safe.

The effect of contextual factors, especially the experience of gender-based violence on girls and young women and how it increases their risk of FGM/C, CEFM and dropping out of school is of great significance. These findings emphasize the need for ARP programmes to be sensitive to other underlying factors that could be preventing complete eradication of the harmful practices that they are trying to address. Ensuring that the ARP programme is well understood and accepted by community members, especially by decision-makers, including parents/guardians, community leaders, church leaders, and school administrators is key in ensuring success (20, 29, 32). This is critical especially for practices such as FGM/C and CEFM where decisions are not merely informed by the choice of an individual or family but dependent on expectations of other families or community members and going against it may negatively affect individual social standing (2, 14).

The findings from this study have programmatic and theoretical implications. Even though data from the 2022 Kenya demographic and health survey show progress at the national level with numerous programmatic interventions implemented at community and grassroots level, FGM/C prevalence rates among the Maasai ethnic group continue to remain relatively high compared to national trends. This study has examined the impact of the CL-ARP model among girls and young women in Kajiado County and demonstrated its potential in protecting girls from FGM/C, CEFM and keeping them in school. The CL-ARP model can be replicated in other similar counties to address FGM/C, CEFM and other practices that negatively affect girls and young women. These findings are equally important in informing the design, implementation and evaluation of ARP interventions in Kenya and other similar settings. Policy makers may also find these findings useful when allocating resources to effective interventions in ending FGM/C in Kajiado County.

Regarding the theoretical implications, findings from this study contribute to the broader theories of social norm change and behavioural interventions (14, 33). The fact that the structure of CL-ARP model emphasizes on a community-led cultural alternative to FGM/C that retains cultural rituals and ceremonies while replacing FGM/C by sexual and reproductive health rights education and the promotion of girls' education could be a starting point towards shifting existing social norms that drive harmful practices such as FGM/C and CEFM. The intergenerational community dialogues with various community members that include men and boys to address social norms, attitudes and behaviours that underly harmful practices and other forms of sexual and gender-based violence have potential to be adapted in other contexts.

## Implications for further research

Given that the study investigated a sensitive and illegal practice such as FGM/C in Kenya using a direct questioning approach, future research could explore using indirect measurement approaches that have been used to measure changes in other stigmatised or sensitive behaviours like FGM/C. Future studies should equally consider collecting data on other external factors that could be responsible to the observed changes in outcome indicators to enable their inclusion in the regression models.

## Strengths and limitations

The strength of this study relates to the longitudinal study design that made it possible to account for the key events and experiences of girls who had participated in the CL-ARP for over three years. Specifically, the longitudinal design enabled the follow-up of girls who had gone through CL-ARP and determine the effect of CL-ARP on FGM/C, CEFM and school attendance. The use of a digital platform to track girls and conduct check-ins was a strength of the study as it facilitated live tracking of girls and provision of a response whenever girls were determined to be at risk. The digital platform also ensured instant and accurate data entry over the years. The other strength relates to the unique findings of the study that have highlighted the effectiveness of the CL-ARP model in helping girls stay free of FGM/C and CEFM, and stay in school to pursue their dreams.

Nevertheless, findings from this study need to be interpreted bearing in mind the following limitations. First, the FGM/C status of girls and young women was self-reported and prone to bias. Even though self-reporting is a valid approach in research especially when dealing with outcomes that cannot be measured through observation, its limitations cannot be ignored in this study. While respondents were reassured of confidentially, anonymity and no implications no matter their responses, there is a possibility that their participation in the CL-ARP programme may have influenced their response with a bias towards stating that they were uncut so that they do not look like failures. Second, the number of months saved from FGM/C, CEFM and school dropouts cannot entirely be attributed to the CL-ARP programme as some of the girls may have already participated in other ARPs before the commencement of check-ins by community health volunteers. Related to the issue of attribution is the fact that external factors such as government policies, media campaigns, increased education and awareness spearheaded by civil society organizations and shifts in global and national attitudes towards FGM/C could have played a role in the observed changes. A look at trends of FGM/C prevalence in Kajiado County shows that there have been a substantial decline from 36% in 2014 to 24% in 2022 (34). Therefore, these external factors need to be acknowledged to avoid over-attribution of the intervention's impact and provide a more nuanced understanding of the observed changes. Third, there is a possibility that some cases of FGM/C, CEFM and girls dropping out of school were not reported. We cannot therefore assume that lack or reporting of cases directly means that the girls are free of FGM/C, CEFM or dropping out of school. Lastly, there was limited variability in outcome variables and therefore the logistic regression models are affected by sparse data bias. Consequently, some of the 95% confidence intervals around the odds ratios in the logistic regression models are very wide pointing to the unreliability of such estimates. Readers should interpret the odds ratios together with the respective 95% confidence intervals for meaningful understanding.

Lastly, we note an adjustment to the study design from a stepped-wedge cluster randomised controlled trial, as initially described in the published protocol, to a longitudinal cohort design without a control group. This change was necessitated by strong resistance from local administration and community members, who expressed ethical concerns about withholding the CL-ARP intervention from a subset of eligible girls, even temporarily. Their position underscored the deep community buy-in and perceived value of the intervention, which we felt it was important to respect. Moreover, given the long history of various forms of ARP implementation within the community, establishing a true control group—unexposed to any form of ARP proved unfeasible. As such, we adapted the design to ensure that all eligible girls were able to access the intervention, while increasing the frequency of follow-up visits to capture key events and outcomes over time. While we acknowledge that a longitudinal design without a control group does not provide the same level of causal inference as a randomised controlled trial, it nevertheless offered an opportunity to observe and document the experiences of girls over an extended period. This design allowed us to capture meaningful trends and outcomes related to FGM/C, child marriage, and school attendance—offering valuable insight into the sustained effects of the CL-ARP intervention.

## Conclusions

The CL-ARP programme managed to keep over 98% of girls and young women free of FGM/C, CEFM and to stay in school after more than three years after they were enrolled into the programme. For girls who either underwent FGM/C, experienced CEFM or dropped out of school, the CL-ARP programme managed to keep them free of FGM/C, CEFM and in school for a substantial period of time. These findings highlight the importance of community-led interventions and their impact on protecting girls and young women from harmful traditional practices such FGM/C and CEFM while keeping them in school. While the CL-ARP model showed impressive results in protecting girls and young women from FGM/C and CEFM and keeping them in school, cases of FGM/C, CEFM and school dropouts were reported. This underlines the importance of considering other contextual factors such as gender-based violence that may still continue to put girls and young women at risk despite embracing CL-ARP.

## Data Availability

The raw data supporting the conclusions of this article will be made available by the authors, without undue reservation.
